# Anesthetic Management of Renal Cell Carcinoma With Level 3 Inferior Vena Cava Extension in a Patient With Severe Coronary Artery Disease

**DOI:** 10.7759/cureus.44180

**Published:** 2023-08-26

**Authors:** Kyle R Gashler, Alan K Ritchie, Ryan Hood, Stephanie O Ibekwe

**Affiliations:** 1 Anesthesiology, Baylor College of Medicine, Houston, USA

**Keywords:** renal cell carcinoma (rcc), coronary artery disease, perioperative evaluation, cardiovascular and thoracic surgery, urologic surgery, multidisciplinary discussion

## Abstract

A 49-year-old male with untreated type 2 diabetes and a family history of coronary artery disease (CAD) presented with right flank pain and profound progressive dyspnea on exertion to the emergency department of Ben Taub Hospital, a tertiary county hospital. Workup revealed right renal cell carcinoma with metastatic extension into the inferior vena cava (IVC). In addition, the patient had significant CAD with 95% occlusion of the proximal left anterior descending coronary artery amenable to percutaneous coronary intervention (PCI). After multidisciplinary discussions involving cardiovascular anesthesiology, cardiology, urology, and cardiothoracic surgery, it was estimated that the mortality benefit of immediate tumor resection outweighed the patient’s need for PCI and further cardiac optimization. The patient underwent curative resection and thrombectomy under transesophageal echocardiography (TEE) guidance without complication, made an expedient recovery, and was discharged home on postoperative day seven.

## Introduction

Renal cell carcinoma (RCC) is the most common renal malignancy, currently accounting for >90% of renal malignancies and totals 2-3% of all adult malignancies in the United States [1]. In about 10% of cases, RCC is complicated by cavoatrial extension, and this finding is associated with increased mortality [2]. Four levels of cavoatrial invasion have been described, with greater levels of invasion requiring more extensive surgical resection [3]. Level 1 thrombus extends to the distal inferior vena cava (IVC) less than 2 cm above the renal vein; level 2 thrombus extends at least 2 cm into the IVC but does not involve the hepatic vein; level 3 thrombus involves the hepatic vein but remains below the diaphragm; level 4 thrombus extends into the supradiaphragmatic IVC or the right atrium. While radical nephrectomy can be curative in patients with RCC without metastatic disease, patients with advanced disease progression due to either delayed diagnosis or delayed treatment experience markedly increased mortality [4].

Given the time-sensitive nature of this potentially curative surgery, anesthesiologists must perform evidence-based risk evaluation and obtain appropriate additional workup to optimize their patients' status without unnecessarily delaying care. We present a case that required us to weigh the benefits of surgical cardiac optimization via percutaneous coronary intervention (PCI) against the timely surgical resection of the tumor. In addition, we discuss the role of PCI in cardiac optimization prior to non-cardiac surgery (NCS).

This case was previously presented at the medically challenging case poster symposium during the 2022 American Society of Anesthesiologists (ASA) Annual Meeting on October 23, 2022.

## Case presentation

A 49-year-old male with a past medical history of untreated type 2 diabetes mellitus (Hemoglobin A1c 7.9), ulcerative colitis, family history of coronary artery disease (CAD), and remote history of gastric lymphoma status post resection and chemotherapy presented to the Ben Taub emergency department with flank pain and increasing shortness of breath for the past year. Three months prior, he was diagnosed with a right renal mass at an outside hospital but was lost to follow-up there. On this subsequent presentation, he was admitted to Ben Taub General Hospital with a plan for expedited workup and potential surgical resection.

**Figure 1:**
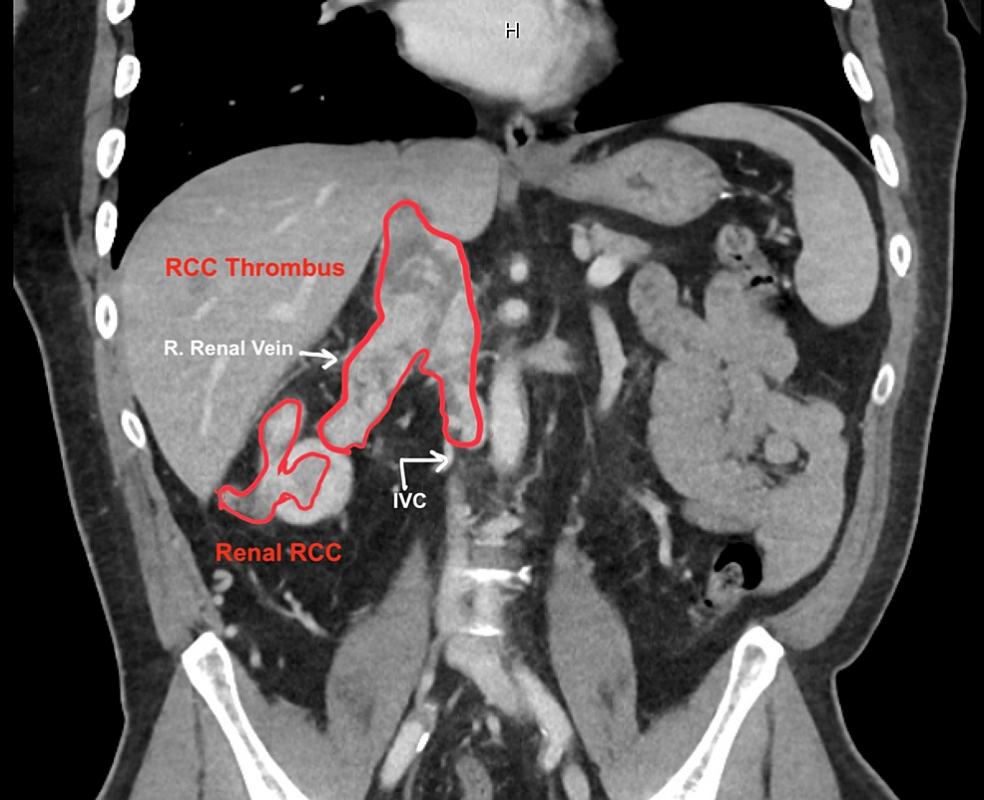
CT abdomen (coronal view) demonstrating right kidney renal cell carcinoma with tumor thrombus in the right renal vein extending to the inferior vena cava. RCC: Renal cell carcinoma; IVC: Inferior vena cava

Labs revealed an initial hemoglobin of 10.4 g/dL and normal chemistries. His baseline EKG was unremarkable. Staging computerized tomography (CT) scans demonstrated right RCC measuring 3.4 x 3.2 x 2.9 cm with level 3 cavoatrial invasion (involving the intrahepatic vena cava but below the diaphragm) with no distant metastases (Figure 1). Given these findings, urology planned for curative resection via open right radical nephrectomy with inferior vena cava (IVC) thrombectomy.

The patient's functional status was limited, with estimated metabolic equivalents (METS) < 4 and profound dyspnea on exertion. The patient's revised cardiac risk index (RCRI) was 1 for elevated-risk surgery, and the American College of Surgeons National Surgical Quality Improvement Program (ACS NSQIP) calculator estimated the risk of cardiac complication to be elevated at 3.3% [5,6]. A transthoracic echocardiogram (TTE) was obtained. The findings were left ventricular ejection fraction greater than 65%, no significant valvulopathy, and no wall motion abnormalities. A nuclear stress test showed a medium-sized area of reversible perfusion defect in the left anterior descending (LAD) coronary artery distribution. Left heart catheterization (LHC) demonstrated severe obstructive coronary artery disease, with 95% stenosis of the proximal to mid LAD. During the LHC, interventional cardiology decided not to intervene with PCI prior to further multidisciplinary discussion.

Several multidisciplinary meetings involving cardiovascular anesthesiology, cardiology, cardiothoracic surgery (CTS), and urology were held to discuss the timing of the nephrectomy and thrombectomy versus PCI. Regarding the patient's RCC, the discussion emphasized the importance of early resection as potentially curative or at least significantly decreasing mortality [4]. Concerning the patient's CAD, our deliberations weighed the risks and benefits of pre-resection PCI and the impact that may have on intraoperative major adverse cardiac events (MACE). PCI has not been shown to reduce perioperative MACE or mortality in patients undergoing NCS [7] -- see further discussion below. We also reviewed the various stents available to our patient, the long-term benefit of each respective stent, and the surgical implications carried by the necessitated post-PCI dual antiplatelet therapy (DAPT) according to American Heart Association (AHA) guidelines [8]. The consensus of the discussion was that the mortality benefit of immediate tumor resection outweighed the patient's need for PCI and further cardiac optimization.

Perioperative management

Given the patient's severe CAD, confounding comorbidities, and extensive tumor burden, an ASA physical status of four was assigned. We had significant concern about the high risk for sudden intraoperative cardiovascular collapse. Pertinent patient and surgical risks included profound hemodynamic changes on induction and maintenance of anesthesia, myocardial infarction, left or right ventricular failure, pulmonary embolism, tumor mass effect on the IVC, acquired coagulopathies, and the potential for significant surgical blood loss. As such, we instituted a multitude of prophylactic safety measures. Preinduction, we placed standard ASA monitors, two large-bore peripheral IVs, and an arterial line. We ensured that crossmatched blood products and a rapid transfuser were immediately available in the operating room.

Additionally, we requested that the CTS team and a perfusionist with a primed cardiopulmonary bypass (CPB) circuit be present in the OR for induction. After a gentle and uncomplicated induction of general anesthesia using a combination of versed, etomidate, propofol, and fentanyl, the patient was intubated, and right internal jugular central venous access was obtained under careful ultrasound guidance. Before incision by Urology, CTS placed prophylactic peripheral vascular access sheaths in case the patient needed emergent peripheral cannulation for CPB.

**Figure 2:**
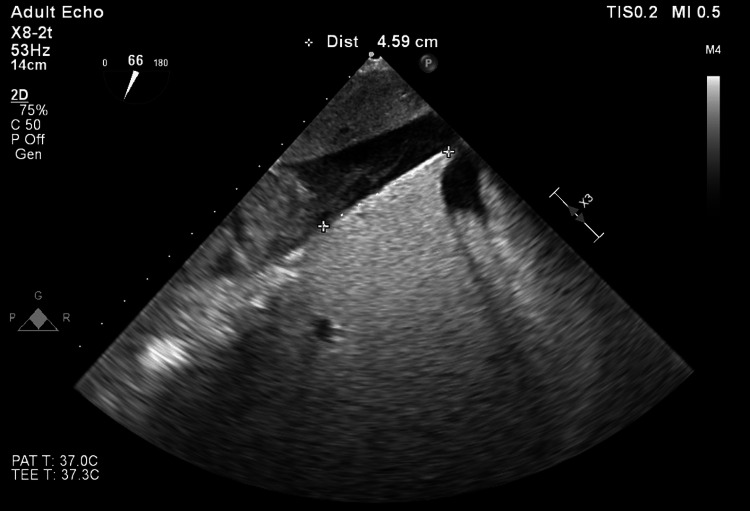
Intraoperative TEE demonstrating IVC thrombus with liver in long axis. TEE: Transesophageal echocardiography; IVC: Inferior vena cava

Intraoperative transesophageal echocardiography (TEE) was performed to guide hemodynamic management, confirm caval tumor thrombus stagging, monitor for possible thrombus migration during manipulation, and guide surgical resection. Hemodynamic goals for the case included ensuring adequate coronary perfusion by maintaining euvolemia, a low-normal heart rate, and a high-normal afterload. The IVC tumor thrombus was visualized approximately 4.6 cm below the junction of the IVC and the hepatic vein (Figure 2).

The nephrectomy and IVC thrombectomy were completed successfully and without complication. Throughout the case, the patient remained hemodynamically stable with minimal vasopressor support. Two units of packed red blood cells were transfused due to intraoperative blood loss. An institutional ERAS multimodal pain management approach was employed using preoperative acetaminophen and tramadol, intraoperative fentanyl, hydromorphone, and ketamine, and postoperative regional anesthesia using combined transverse abdominal (TAP) and quadratus lumborum (QL) blocks. The patient was monitored postoperatively in the surgical ICU, where he was successfully extubated on postoperative day 1. A follow-up CT scan showed that the patient was tumor free with a patent but diminutive IVC. The remainder of his hospital course was uneventful, and he was successfully discharged home on postoperative day 7.

The patient was scheduled for follow-up PCI to address his LAD lesion several weeks after his RCC resection. However, the patient was again lost to follow-up until five months later when he presented to the Ben Taub emergency department with unstable angina. The patient underwent a successful two-vessel coronary artery bypass during that admission.

## Discussion

Assessment of a patient's cardiac status and risk of a perioperative Major Adverse Cardiac Event (MACE) is important in every anesthetic pre-evaluation. In addition to a focused history and physical and an estimation of functional capacity (i.e., METS), AHA guidelines endorse the use of scoring systems such as the RCRI or the ACS NSQIP to evaluate cardiac risk [8].

Due to the patient's poor functional capacity (< 4 METS), our patient underwent pharmacologic stress testing followed by coronary angiography, ultimately revealing severe CAD amenable to PCI. However, the role of PCI prior to NCS in reducing MACE is uncertain. To date, no prospective randomized controlled trial has demonstrated a reduction in MACE or mortality in patients who underwent PCI prior to NCS [7]. The Coronary Artery Revascularization Prophylaxis (CARP) trial demonstrated that coronary revascularization with coronary artery bypass grafting (CABG) or PCI prior to elective vascular surgery did not improve perioperative outcomes or long-term mortality [9]. Retrospective data have even suggested that PCI before high-risk NCS may increase the risk of MACE [8].

In addition, PCI has known risks that must be considered, and the necessitated course of post-PCI DAPT creates its own challenges. After PCI, the AHA recommends a stent-specific duration and formulation of DAPT [10]. If DAPT is continued through the perioperative period, there is a profound risk for surgical bleeding and coagulopathy in the setting of iatrogenic platelet inhibition. Alternatively, if DAPT is discontinued preemptively, the patient is now exposed to the risk of in-stent thrombosis, an otherwise potentially avoidable ischemic event. Thus, any consideration of non-emergent PCI prior to NCS should be limited to a highly vetted patient population after a thorough multidisciplinary discussion balancing the immediate risks and benefits of each respective intervention considered for the patient.

## Conclusions

Multidisciplinary planning and communication were crucial to the success of this complex, high-risk surgical procedure. Preoperative evaluations should be evidence-based to obtain an appropriately thorough workup. A comprehensive workup ensures the best possible patient outcomes without unnecessary delays in care. In our case, when the patient’s workup revealed that the patient urgently needed both PCI and tumor resection, multidisciplinary discussions were required to develop a comprehensive and safe plan for the patient. Specifically, understanding that coronary revascularization immediately prior to NCS does not decrease, and may even increase, the risk of MACE was key in our decision to proceed with tumor resection in an otherwise cardiovascularly unoptimized patient.

When creating the anesthetic plan for a patient, it is vital to understand each patient's disease pathology and how that will impact their physiologic demands during surgery. Ensuring sufficient vascular access, the availability of rapid transfusion equipment, and having cross-matched blood products ready in the operating room is imperative when massive blood loss is imminent. TEE continues to prove invaluable for both surgical guidance and managing intraoperative hemodynamics. Lastly, preparation for cardiovascular collapse and the possibility of emergent CPB is imperative when cardiac optimization cannot be achieved.
